# Ozone-Induced Hypertussive Responses in Rabbits and Guinea Pigs

**DOI:** 10.1124/jpet.115.230227

**Published:** 2016-04

**Authors:** Emlyn Clay, Riccardo Patacchini, Marcello Trevisani, Delia Preti, Maria Pia Branà, Domenico Spina, Clive Page

**Affiliations:** Sackler Institute of Pulmonary Pharmacology, Institute of Pharmaceutical Science, King’s College London, London, United Kingdom (E.C., D.S., C.P.); Department of Corporate Drug Development (R.P.), and Department of Pharmacology (M.T.), Chiesi Farmaceutici SpA, Parma, Italy; Department of Chemical and Pharmaceutical Sciences, University of Ferrara, Ferrara, Italy (D.P.); and Department of Health Sciences, Section of Clinical Pharmacology and Oncology, University of Florence, Florence, Italy (M.P.B.)

## Abstract

Cough remains a major unmet clinical need, and preclinical animal models are not predictive for new antitussive agents. We have investigated the mechanisms and pharmacological sensitivity of ozone-induced hypertussive responses in rabbits and guinea pigs. Ozone induced a significant increase in cough frequency and a decrease in time to first cough to inhaled citric acid in both conscious guinea pigs and rabbits. This response was inhibited by the established antitussive drugs codeine and levodropropizine. In contrast to the guinea pig, hypertussive responses in the rabbit were not inhibited by bronchodilator drugs (*β*_2_ agonists or muscarinic receptor antagonists), suggesting that the observed hypertussive state was not secondary to bronchoconstriction in this species. The ozone-induced hypertussive response in the rabbit was inhibited by chronic pretreatment with capsaicin, suggestive of a sensitization of airway sensory nerve fibers. However, we could find no evidence for a role of TRPA1 in this response, suggesting that ozone was not sensitizing airway sensory nerves via activation of this receptor. Whereas the ozone-induced hypertussive response was accompanied by a significant influx of neutrophils into the airway, the hypertussive response was not inhibited by the anti-inflammatory phosphodiesterase 4 inhibitor roflumilast at a dose that clearly exhibited anti-inflammatory activity. In summary, our results suggest that ozone-induced hypertussive responses to citric acid may provide a useful model for the investigation of novel drugs for the treatment of cough, but some important differences were noted between the two species with respect to sensitivity to bronchodilator drugs.

## Introduction

Cough remains a significant unmet medical need, and there have been no new effective drugs introduced for this common symptom for several decades (Dicpinigaitis et al., 2014). Furthermore, many of the existing drug classes for treating cough are considered ineffective or have unacceptable side effects, and there is therefore a clear need for the development of novel antitussive drugs (Dicpinigaitis et al., 2014). A significant challenge in the discovery and development of new antitussive drugs is the lack of robust and predictive nonclinical models (Belvisi and Bolser, 2002; Mackenzie et al., 2004). To date, a range of species have been investigated as potential models of the cough associated with a range of clinical conditions, including pigs, horses, cats, guinea pigs, and rabbits (Jackson, 1988; Schelegle et al., 2001; Mackenzie et al., 2004; Ferrari et al., 2009; Duz et al., 2010). Many of these involve the investigation of the effect of drugs against cough elicited by tussive challenges, such as citric acid and capsaicin, in an attempt to mimic the use of these tussive stimuli in the clinical setting, for both the evaluation of cough sensitivity and the effectiveness of novel treatments.

A number of drugs, including thalidomide (Horton et al., 2012), gabapentin (Ryan et al., 2012), morphine (Morice et al., 2007), and a P2X3 receptor antagonist (Abdulqawi et al., 2015), but not a transient receptor potential cation channel subfamily V member 1 (TRPV1) antagonist (Khalid et al., 2014), have recently been shown to suppress chronic cough in humans. Interestingly, however, morphine did not significantly alter cough sensitivity to citric acid despite reducing cough in patients with chronic cough (Morice et al., 2007), whereas in contrast, the TRPV1 antagonist SB-705498 (1-(2-bromophenyl)-3-[(3R)-1-[5-(trifluoromethyl)pyridin-2-yl]pyrrolidin-3-yl]urea) suppressed cough in response to inhaled capsaicin, yet showed no clinical benefit in reducing cough in patients with chronic cough (Khalid et al., 2014). These clinical findings question the validity of using citric acid or capsaicin challenge as a surrogate marker for spontaneously elicited cough in diseased airways. However, almost all preclinical models involve the investigation of cough challenges in healthy animals, even though the drugs under investigation are being developed to treat cough in patients who exhibit a heightened cough reflex termed a hypertussive response or cough hypersensitivity (Canning et al., 2014; Morice et al., 2014). In fact, it is questionable whether there is really a need for an antitussive drug; rather, what is required is a drug to reduce this hypertussive state that otherwise permits inappropriate or exaggerated cough responses, often to stimuli that do not normally induce coughing in healthy people.

It is now appreciated that this hypertussive state may be analogous to hyperalgesia or allodynia (O'Neill et al., 2013), and as such, there may be much to learn from the field of pain research as to how we might improve our selection of targets for new antitussive agents and improve the usefulness of our nonclinical models. We have previously described a method for establishing hypertussive responses in the rabbit following acute exposure to an inhalation of ozone, but little is known about the mechanisms underlying this hypertussive state or the pharmacological sensitivity of this model (Adcock et al., 2003). In the present study, we further investigated this model in the rabbit and extended our work to also investigate ozone induced hypertussive responses in the guinea pig, a species often used in pulmonary pharmacology.

## Methods

### 

#### Animals.

Male New Zealand white rabbits (2.5–4 kg) were supplied from the Biologic Services Unit, Northwick Park Hospital (London, UK). Male Dunkin-Hartley guinea pigs (300 to 500 g) were supplied by Harlan (Oxfordshire, UK). The animals were housed in the biologic services unit of King’s College London (London, UK), with a 16-hour day and 8-hour night cycle. Food and water were accessible ad libitum and routinely checked by the biologic science unit’s technical staff. All studies in animals were carried out in accordance with the Operation of Animals Scientific Procedures Act of the United Kingdom, and were approved by the local ethics committee of King’s College London.

#### Drug Preparation.

Citric acid monohydrate, acetyl-*β*-methylcholine chloride, salbutamol sulfate, atropine, cinnamaldehyde, codeine phosphate, chlorpheniramine maleate (Sigma-Aldrich, Poole, UK), and levodropropizine, (Eurodrug, The Hague, Netherlands), were all dissolved in sterile saline (0.9%; Baxter Healthcare Ltd., Thetford, UK). Tiotropium bromide (Chiesi Farmaceutici, Parma, Italy) was prepared as a stock solution of 6 mM and diluted with sterile saline. All drugs were prepared fresh on the day of use. Roflumilast (CAS162401-32-3; Kemprotec, Carnforth, UK) is poorly soluble in saline and was therefore prepared in neat solutol (2%, Kolliphor HS15, 42966; Sigma-Aldrich) and diluted to the final concentration on the day of use. Capsaicin (Sigma-Aldrich) was prepared as a stock solution of 25 mg/ml (8:1:1 saline:ethanol:Tween 20; Fisher Scientific, Loughborough, UK). HC-030031 (2-(1,3-dimethyl-2,6-dioxo-1,2,3,6-tetrahydro-7H-purin-7-yl)-*N*-(4-isopropylphenyl)acetamide; Chiesi Farmaceutici) was prepared as a stock solution of 300 mg/ml in sterile saline and 1% solutol.

#### Measurement of Cough.

**Fig. 1.**
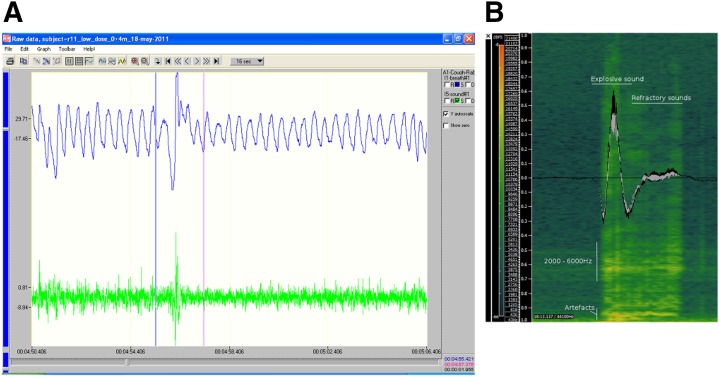
(A) Screenshot of the simultaneous change in pressure and sound that EMKA uses to classify a cough event. The cough shown was provoked by exposing a rabbit to aerosolized citric acid. (B) Screenshot of Sonic Visualizer and how the audio signal and frequency domain were manually observed to determine a cough. This cough response was provoked by exposing a rabbit to aerosolized citric acid.

Coughs elicited by rabbits and guinea pigs were classified using a cough analyzer (EMKA Technologies S.A.S., Paris, France). The system comprised a Perspex whole-body plethysmograph chamber with a single microphone (EMKA) attached to an amplipower sound amplifier (PS100W-Z; EMKA) and a pressure transducer (EMKA) attached to a separate amplipower amplifier (DS100W; EMKA). In the case of the rabbit, a custom-built Perspex chamber was used for ozone exposure and registration of cough. In the case of guinea pigs, animals were exposed to ozone within the custom-built Perspex chamber, then transferred to the EMKA cough chambers. In both cases, the interval between cessation of ozone exposure and commencement of exposure to tussive agents was 20 minutes. The output from both amplifiers was connected to a data acquisition card on a personal computer (NI PCI-6255; National Instruments, Austin, TX), and the data were recorded and observed using the I.O.X. software (v1.7.1; EMKA). The number of coughs and sneezes was counted during the 10-minute dosing period with inhaled citric acid (rabbit, 0.4 and 0.8 M; guinea pig, 30 and 100 mM) and the 5-minute postdose period. Coughs were classified as the simultaneous increase of the pressure and sound rising sharply above a threshold and back to baseline within 500 ms (Fig. 1A). The thresholds were determined in initial experiments.

In addition, coughs in the rabbit were classified by listening to the sound that was made, as well as observing characteristic frequency bands between 3500 and 6000 Hz in the temporal components of the cough (Fig. 1B). Cough events were monitored using the audio recording and analyzed using the open-source Sonic Visualizer software (open source: (http://www.sonicvisualiser.org) to allow appropriate differentiation of coughs from sneezes and to measure the length and amplitude of each cough and sneeze sound.

#### Ozone Exposure.

Rabbits were placed unrestrained in a custom-built Perspex chamber and allowed to acclimatize for 15 minutes, and were then exposed to ozone or air for 1 hour. Ozone was generated by passing air through an ozonizer (Certizon C25; Sander, UK) at a flow rate of 5 l/min, and the exhaust air from the chamber was measured in real time using an electronic ozone sensor (Aeroqual 200 series; Aeroqual Ltd., UK). An analog dial controlled the strength of ozonizer output, and this was adjusted, using the electronic sensor as a measure, to achieve a target atmosphere of 2 ppm in the chamber.

Guinea pigs were placed in a Perspex chamber, 2–4 at a time, and exposed to a target atmosphere of 2 ppm of ozone. However, an unacceptable rate of cyanosis resulted from using the same exposure protocol for both rabbits and guinea pigs, the exposure time was reduced from 1 hour to 30 minutes in guinea pigs while maintaining the same target atmosphere.

#### Pulmonary Lung Mechanics.

Rabbits were anesthetized with a mixture of ketamine (34 mg/kg; Vetalar, 100 mg/ml; Animal Health, Pfizer Ltd, Sandwich, UK), and xylazine (20 mg/kg; Rompun, 23.32 mg/ml, 2%, Animal Health Division, Bayer plc, Newbury UK) administered intramuscularly and, if required, maintenance anesthesia (50% then 25% at 20-minute intervals) until adequate anesthesia was obtained. This anesthetic regimen was chosen so that total lung resistance (R_L_) and dynamic lung compliance (Cdyn) could be monitored noninvasively and repeatedly in rabbits without the need for artificial ventilation (Keir et al., 2011).

An endotracheal tube (i.d. 3.0, Covidien, Whiteley, United Kingdom) was inserted into the trachea and cuff inflated once in position. An esophageal balloon was then inserted. The rabbit was then transferred onto a heating mat (Harvard Apparatus, Natick, MA), and a temperature probe was inserted into the rectum to thermostatically maintain the core body temperature at around 37°C. The endotracheal tube was attached to a heated pneumotachograph (type 00) connected to a pressure transducer (MuMed BR8101, S/N 960303, ±2 cm of water; Mumed Systems Ltd., London, UK) to obtain a measure of airflow. The esophageal balloon was connected to the negative side of the pressure transducer (Mumed BR8101, S/N 960338, ±20 cm of water; Mumed Systems Ltd.) to obtain a measure of intrapleural pressure. The positive side of the pressure transducer (Mumed BR8101, S/N 960338, ±20 cm of water; Mumed Systems Ltd.) was connected to the port of the pneumotachograph proximal to the animal to obtain a measure of mouth pressure. Transpulmonary pressure was calculated as the difference between the mouth and intrapleural pressure. An online recording of airflow, tidal volume, and TPP (bio-recorder BR8000; Mumed Systems Ltd.) was used to calculate breath by breath, R_L_ and C_dyn_. Baseline variables (R_L_, C_dyn_) were monitored over a 5- to 8-minute period. Rabbits were then exposed for 20 seconds to 0.9% saline by disconnecting the endotracheal tube from the pneumotachograph and attaching it to the tube of the nebulizer (UltraNeb 2000; DeVilbiss Healthcare Ltd., Tipton, UK) to deliver aerosols of saline directly to the lung. Rabbits breathed nebulizer solutions spontaneously, and a side arm to the nebulizer tube permitted breathing under atmospheric conditions. The endotracheal tube was reattached, and changes in R_L_ and C_dyn_ were monitored and allowed to reach a steady state. Once this steady state was achieved, the endotracheal tube was once again disconnected, and dosing with methacholine was commenced. This cycle was repeated, escalating the dose until a maximum response was achieved. The dose of methacholine delivered ranged from 0.30 to 80 mg/ml over a period of 20 seconds of exposure. Postanalysis was undertaken to calculate the dose that caused a doubling in either respiratory rate or R_L_ or a halving of C_dyn_. The recording software ran continuously throughout the experiment.

In other experiments, guinea pigs were terminally anesthetized with urethane (25%; Sigma-Aldrich) by the intraperitoneal route given in decrementing doses four times every 30 minutes for 2 hours. The dosage started at 2 g/kg, then 1 g/kg (1/2 of the original dose), and then 0.5 g/kg (1/3 of the original dose) until adequate anesthesia was achieved. Guinea pigs were tracheostomized, and a cannula was inserted (1.65 mm i.d.) into the lumen of the cervical trachea, tied in place, and connected via a pneumotachograph to a ventilator. A cannula was inserted between the fourth and fifth intercostal rib to measure intrathoracic pressure. Guinea pigs were mechanically ventilated in the supine position by a constant-volume ventilator (model 683; Harvard Apparatus) at 8 ml/kg tidal volume and a frequency of 60 breaths/min to permit the recording of R_L_ and C_dyn_. A jugular vein was cannulated for the intravenous administration of drugs. The dose of methacholine delivered ranged from 0.04 to 1.25 mg/ml over a period of 20 seconds of exposure.

#### Enumeration of Inflammatory Cells.

A bronchoalveolar lavage (BAL) was performed following completion of lung function in anesthetized rabbits. A cannula was inserted within the endotracheal tube, and 5 ml of sterile saline was administered into the lung followed by recovery of the fluid under gentle vacuum suction. The fluid was left in the lung for no more than a few seconds, and the amount of fluid recovered was noted (approximately 60% recovery). An aliquot of the lavage fluid (50 *μ*l) was fixed with 50 *μ*l of Turk’s solution (catalog number 109277; Merck KGaA, Darmstadt, Germany). Total cell counts were performed for this solution using a Neubauer hemocytometer. Two 100 *μ*l samples of neat BAL fluid were centrifuged (Cytospin 3 Centrifuge; Thermo Scientific Shandon, Waltham, MA) onto a glass slide. The slides were left to dry, fixed (Reastain Quick-Diff Kit; Reagena, Toivala, Finland), and then mounted in DPX Mountant (Sigma-Aldrich). Differential cell counts were performed using confocal microscopy with oil at a 40× magnification.

#### Experimental Design for Lung Function Postozone Challenge.

Sixteen rabbits and 32 guinea pigs were investigated. Each animal was “screened” on day 1 by exposure to citric acid (0.8 M for rabbits and 30 mM for guinea pigs) prior to ozone challenge or drug treatment, and the animals’ baseline sensitivity to citric acid was established. On day 7, the animals randomly received ozone sensitization or air exposure, and then on day 14, the animals were crossed over. Four rabbits were crossed over again to establish the repeatability of the ozone-sensitized response. The interval of 7 days between each experiment was used to avoid any possible potentiation of the ozone-sensitized response from chronic exposure.

Seven days after the last cough experiments were finished, lung-function experiments were performed. On day 1 of lung-function experiments, groups of animals were randomized to receive either air or ozone (rabbits, 2 ppm, 1 hour; guinea pigs, 2 ppm, 30 minutes). Four hours later, lung-function experiments began. Animals were anesthetized and intubated, then methacholine was delivered in successive, increasing doses until a doubling of R_L_ and a halving of C_dyn_ was achieved. Rabbits were allowed to recover from anesthesia and underwent a second lung-function experiment 3 days later, so that each rabbit was individually controlled. At the conclusion of each lung-function experiment, a BAL was performed and a total and differential cell count was taken. At the completion of the experiment, animals were humanely killed with an overdose of pentobarbitone (Euthanal; Merial Animal Health Ltd, Harlow, UK).

#### Experimental Design for the Investigation of Drugs on Hypertussive Response Postozone Challenge.

Eight rabbits and 16 guinea pigs were used in a randomized crossover design to evaluate the antitussive effect of salbutamol, roflumilast, and codeine. Each animal was screened on day 1 by exposure to citric acid (0.8 M for rabbits and 30 mM for guinea pigs) prior to ozone challenge or drug treatment to establish the baseline sensitivity of the animal to citric acid. On day 7, the animals were randomly allocated to one of five conditions. Animals were exposed to air, ozone, or ozone prior to drug treatment, and then cough was measured in response to citric acid. Every 7 days thereafter, the same group of animals were crossed over to one of the five conditions. Hence, all animals experienced each condition over five periods at weekly intervals.

Animals were treated with salbutamol (guinea pigs: 50 *μ*g/ml; rabbits: 100 *μ*g/ml, aerosol) 5 minutes prior to citric acid challenge and for a duration of 5 minutes. Roflumilast (1 mg/kg, i.p.) or codeine (3 mg/kg, i.p.) was administered 5 minutes prior to ozone exposure in both species. Seven days after completion of the cough experiments, the dose of salbutamol and roflumilast was validated in lung-function experiments (salbutamol; four rabbits, eight guinea pigs) and against inflammatory cell recruitment (roflumilast; four rabbits), respectively, as previously described.

In other experiments, levodropropizine (10, 30 mg/kg, i.p.) and chlorpheniramine (50 mg/kg, i.p.) alone and in combination (levodropropizine 10 mg/kg/chlorpheniramine 50 mg/kg and levodropropizine 30 mg/kg/chlorpheniramine 50 mg/kg, i.p.) were administered 5 minutes prior to ozone exposure in guinea pigs. A cross-over design was used to evaluate these six treatment conditions in each animal at weekly intervals.

#### Effect of Muscarinic Receptor Antagonists on Hypertussive Responses Induced by Ozone.

After the initial screening, eight rabbits and 16 guinea pigs were randomized to receive tiotropium bromide (250 *μ*M, 10 minutes) by aerosol 2 hours prior to air or ozone exposure to establish the effect of tiotropium bromide on the normotussive and hypertussive cough using the EMKA cough system. A group of ozone-exposed animals received vehicle (saline 0.9%). Seven days after completion of the cough experiments, rabbits were used for lung-function experiments to establish the effect of tiotropium bromide on methacholine-induced bronchospasm in exactly the same protocol that was used to assess the effect of ozone on lung function, but animals received tiotropium bromide before ozone sensitization using the same dose and time of exposure as per the cough studies. BAL was also performed for the enumeration of total and differential cell counts.

#### Capsaicin Desensitization.

Rabbits were treated with capsaicin (total dose 80 mg/kg, s.c.) over a 3-day period (day 1: 0.3, 0.6, 1.5, and 2.6 mg/kg; day 2: 5, 10, 15, 20, and 25 mg/kg; day 3: 25 mg/kg). Rabbits received a premedication cocktail 15 minutes prior to injection with capsaicin (theophylline: 2 mg/kg; atropine: 1.2 mg/kg; diphenhydramine: 2.5 mg/kg; and chlordiazepoxide: 1.2 mg/kg, i.p.). Cough was measured in response to citric acid 2 days after the last capsaicin injection. We have previously shown that this treatment protocol causes a functional desensitization of sensory nerves in airways (Spina et al., 1991).

#### TRPA1 and Hypertussive Cough.

Each animal was “screened” on day 1 by exposure to citric acid (0.8 M for rabbits and 30 mM for guinea pigs) prior to ozone challenge or drug treatment that established the baseline sensitivity of the animal to citric acid. On day 7, rabbits were randomly allocated for exposure to air or ozone and were crossed over to receive these challenges on day 14. On day 21, animals received a 10-minute exposure to aerosolized cinnamaldehyde (800 mM) at a flow rate of 3 l/min, identical to the exposure protocol for citric acid. On day 28, the experiment was repeated with an exposure dose of 1.6 M, and on day 35, the experiment was repeated with an exposure dose of 30 M. Finally, on day 42, the rabbits were sensitized with ozone exposure and then received a 10-minute exposure to aerosolized 30 M cinnamaldehyde at a flow rate of 3 l/min.

Guinea pigs received a 15-minute exposure to aerosolized 30 mM cinnamaldehyde at a flow rate of 3 l/min on day 7. On day 14, the experiment was repeated with an exposure dose of 800 mM cinnamaldehyde. On day 21, guinea pigs were treated with HC-030031 (300 mg/kg, i.p.) 1 hour before aerosol challenge with cinnamaldehyde (800 mM). Finally, on day 28, the guinea pigs were treated with HC-030031 (300 mg/kg, i.p.); then 30 minutes later, they were sensitized with ozone exposure for 30 minutes and then received a 10-minute exposure to aerosolized 30 mM citric acid at a flow rate of 3 l/min.

#### Statistics.

To classify audio events, such as cough and sneeze events, Sonic Visualizer (Chris Cannam and Queen Mary University, London, UK) was used to listen to each individual event, which was then annotated as a cough or a sneeze. The signal was annotated using Sonic Visualizer by manually drawing a small rectangle around each sound event on an annotations layer within Sonic Visualizer; this meant that the start, end, peak, and trough of the event were measured. These measurements were held in an annotation layer, in an XML format.

The annotation layers were analyzed using the Python programming language (https://www.python.org) to script routines to calculate parameters. The width or the interval of an audio event was the difference between the end frame and start frame of an interval and was standardized to seconds; the height was the difference between the absolute peak and the absolute trough and was standardized to volts. The power of the sound event was calculated in a facile manner by multiplying the width of the event by the height of the event.

The response to methacholine was expressed as a percent increase (R_L_) or decrease (C_dyn_) of the postsaline values. The provocative concentration (PC) of methacholine with 50% increase in baseline RL (RL PC50) and 35% decrease in baseline dynamic compliance (C_dyn_ PC35) was used as a measure of sensitivity.

Data are expressed as the mean ± S.E.M. or ± 95% confidence interval (CI). For each pairwise comparison, a paired Student’s *t* test was used, and a *P* value <0.05 was considered significant. For multiple group design, analysis of variance (ANOVA) followed by an appropriate post-hoc test was used. Repeat-measures ANOVA was used when animals received multiple doses and for all crossover designs using a Greenhouse-Geisser correction when sphericity failed, followed by a post-hoc test.

In addition, for crossover designs that involved a drug treatment, a mixed-effect model was performed before the repeat-measures ANOVA to determine whether interactions between the treatment (random), sequence (random), or period (fixed) caused confounding carryover effects on the cough response. The expectation-maximization algorithm was used to fit the parameters of the model. In addition, the treatment groups were balanced to control for the effect of first-order carryover effects, and subjects were randomly assigned a sequence using a random number generator. The mixed-effect model was calculated using the MixedLM function from the statsmodels package in the Python programming language. The parameters for the MixedLM functions were inputted such that cough, the dependent variable, was grouped by the treatment received, and interactions between cough and sequence and cough and period were calculated.

## Results

**Fig. 2.**
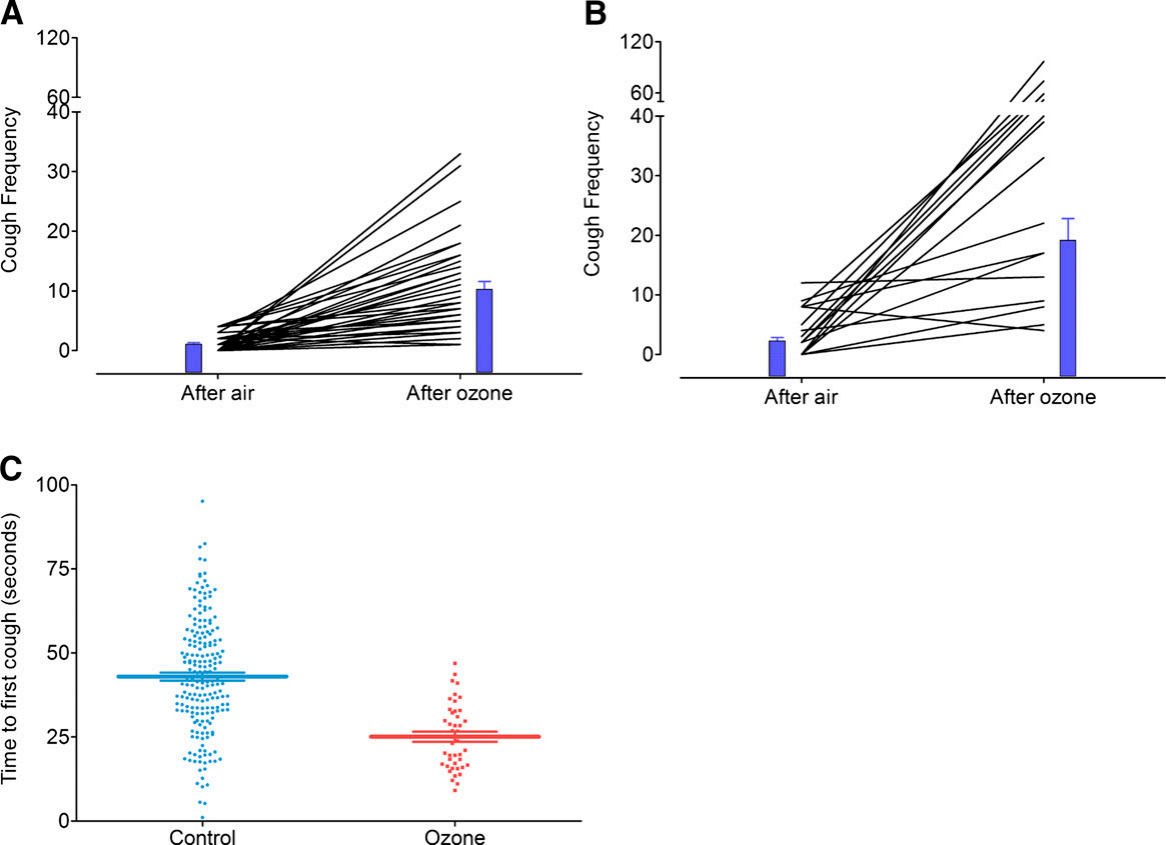
Frequency of cough in response to citric acid (30 mM) (A) and citric acid (100 mM) (B) following exposure to air or ozone (2 ppm, 30-minute exposure) in guinea pigs. The time period between air and ozone exposure was 7 days, and the sequence of whether a guinea pig received ozone or air first was randomized. The blue bars indicate the mean cough response, and the error bars represent the S.E.M. (*N* = 40 and 16, respectively). (C) Time to first cough in guinea pigs, defined as the time in seconds when the first cough occurred after the nebulizer containing citric acid was turned on. The thick horizontal bar represents the mean and the error bars are the S.E.M., N of 198 for the guinea pig controls and 48 for the ozone-sensitized guinea pigs.

In guinea pigs, the cough frequency in response to citric acid (30 and 100 mM) was significantly increased (delta mean, 95% CI) following exposure to ozone compared with exposure to air by 9.25 (6.785–11.72, *P* < 0.008) and by 29.38 (14.33–44.42, *P* < 0.0001), respectively (Fig. 2, A and B), indicating that the response was dose-dependent. The time to cough (mean, 95% CI) in response to citric acid (either dose) was 43.0 seconds (40.6–45.4) in air-exposed animals, which was significantly reduced to 25.1 seconds (22.02–28.18) in the ozone-sensitized group (*P* < 0.0001; Fig. 2C).

**Fig. 3.**
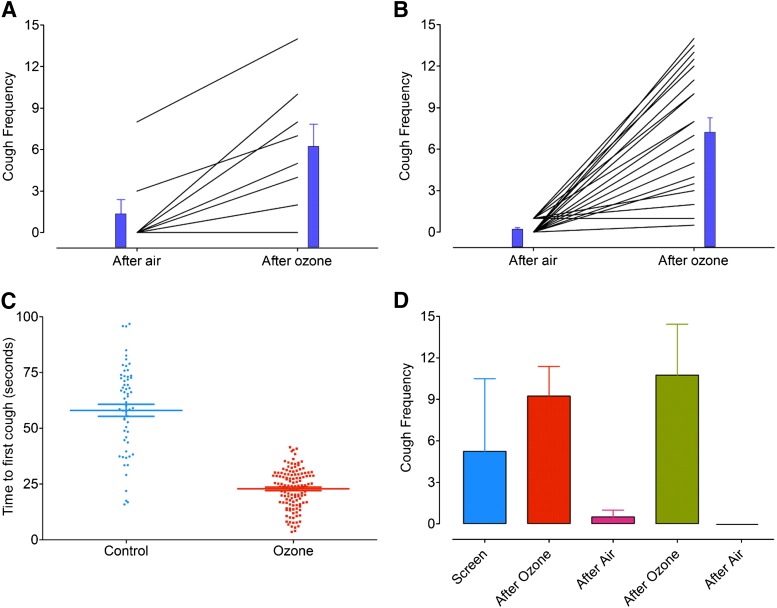
Frequency of cough in response to 0.4 M citric acid (A) and 0.8 M citric acid (B) following exposure to air or ozone (2 ppm, 1-hour exposure) in rabbits. The time period between each group, after air or after ozone, was 7 days, and the sequence of whether a rabbit received ozone or air first was randomized. The blue bars indicate the mean response after air and after ozone, and the error bars represent the S.E.M. (*N* = 8 and 25, respectively). (C) Time-to-first-cough rabbits, defined as the time in seconds when the first cough occurred after the nebulizer containing citric acid was turned on. The thick horizontal bar represents the mean and the error bars are the S.E.M., *N* = 48 for the rabbit controls and *N* = 129 for the ozone-sensitized rabbits. (D) The effect of a repeated challenge with ozone on cough response was measured in a small cohort of rabbits (*N* = 4). Rabbits were challenged with citric acid on day 0, then exposed to either ozone or air on day 7. Rabbits were then crossed over to treatment on day 14, and this crossover by exposure was repeated on days 21 and 28. Column represents the mean and vertical lines represent S.E.M.

In rabbits, the cough frequency in response to citric acid (0.4 and 0.8 M) was significantly increased (delta mean, 95% CI) following exposure to ozone compared with exposure to air by 4.86 coughs (2.21–7.54, *P* = 0.010) and by 6.88 coughs (5.12–8.64, *P* = 0.007), respectively (Fig. 3, A and B). The time to cough (mean, 95% CI) in response to citric acid (either dose) was 58.1 seconds (52.7–63.5) in air-exposed animals, which was significantly reduced to 22.9 seconds (21.4–24.4) in the ozone-sensitized group (*P* < 0.001). Ozone also sensitized rabbits to cough spontaneously without citric acid as a tussive stimuli on one or more occasions. Hence, 74% of the rabbits studied (38 out of 52 rabbits) coughed spontaneously an average of 16 coughs (7.15–20.51, 95% CI) during the 1-hour ozone-sensitization period.

The hypertussive cough response following ozone exposure appears to be greater in guinea pigs compared with rabbits. However, the time to cough to citric acid was not significantly different between species following exposure to air or ozone (*P* > 0.05).

Four rabbits previously exposed to citric acid (0.8 M) were crossed over, and the tussive response to inhaled citric acid was reproducible (Fig. 3D) over time, and the magnitude of the hypertussive response was not significantly different between day 7 and day 14. Furthermore, there was no evidence that repeated ozone challenge caused long-term sensitization of the airways to citric acid (Fig. 3D).

### 

#### The Effect of Muscarinic Receptor Antagonist on the Hypertussive Response to Ozone in Guinea Pigs.

**Fig. 4.**
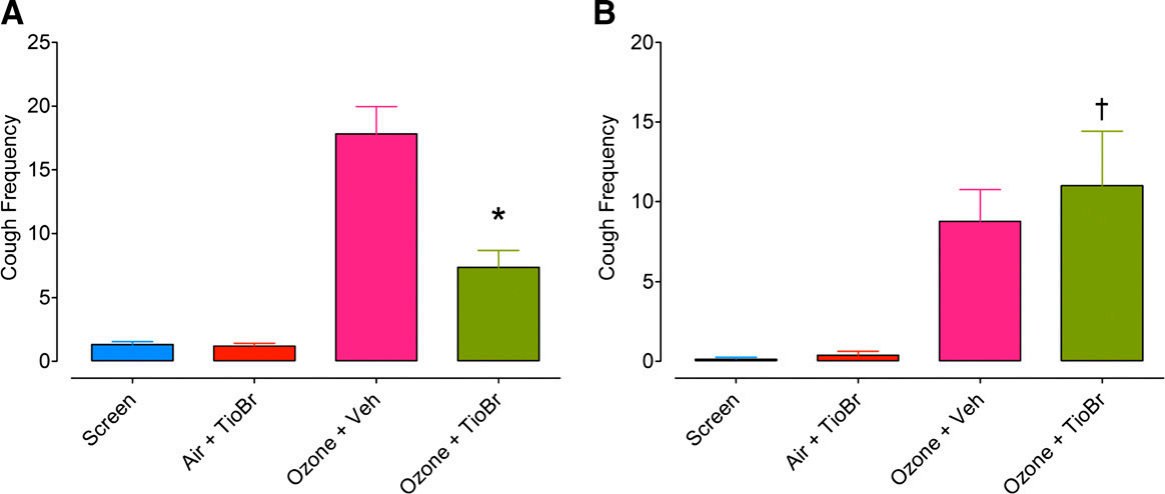
Frequency of cough in response to citric acid in guinea pigs (30 mM) (A) and rabbits (0.8 M) (B) exposed to air or ozone (2 ppm, 30 minutes and 1 hour, respectively) 4 hours after treatment with tiotropium bromide (TioBr, 250 *μ*M; 10 minutes, guinea-pig, *N* = 16; rabbit, *N* = 8) (A and B). The treatment an animal received at 7-day intervals was randomized to control for sequence effects. Bars represent the mean and vertical lines, S.E.M. (*P < 0.01, †*P* > 0.05, compared with ozone/vehicle group).

Guinea pigs at screen or exposed to air following treatment with tiotropium bromide were generally unresponsive to citric acid (30 mM), and this dose was chosen to evaluate the effect of tiotropium bromide on ozone-induced sensitization of the cough response. As we have previously shown, the frequency (mean, 95% CI) of cough increased at screening from 1.31 (0.84–1.78) to 17.8 (13.2–22.4) following ozone exposure [mean difference (95% CI): 16.5 coughs (12.1–20.9), *N* = 16, *P* < 0.0001]. The frequency of cough in guinea pigs exposed to tiotropium bromide (250 *μ*M aerosol for 10 minutes at 3 l/min) 2 hours prior to ozone exposure was reduced to 7.38 (4.63–10.1). Hence, there was a significant reduction in the number of coughs [delta change = 10.3 (5.4–15.2), *P* = 0.0005, *N* = 16] following treatment with tiotropium bromide (Fig. 4A).

The mean (95% CI) frequency of cough significantly increased in rabbits exposed to ozone [screen: 0.12 (0.017–0.42) compared with ozone/vehicle: 8.75 (4.03–13.5), *P* < 0.01, Fig. 4B]. This mean increase in frequency of cough was not attenuated by treatment with tiotropium bromide [delta change = −2 (−11.1–7.1), *N* = 8, *P* > 0.05, Fig. 4B].

#### Effect of Ozone on Airway Responsiveness to Methacholine.

**Fig. 5.**
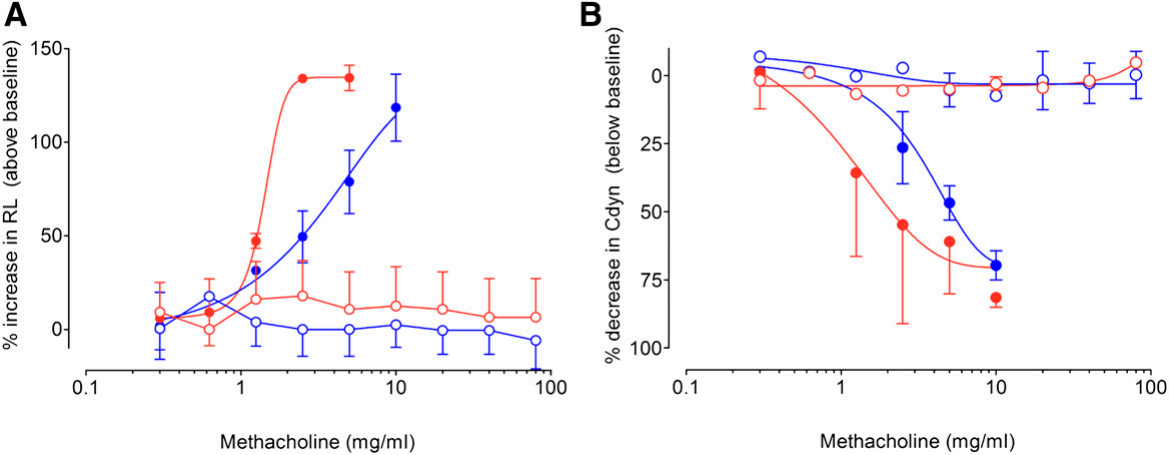
Cumulative dose-response curves to aerosolized methacholine in the absence of (closed symbols) or following treatment with muscarinic antagonists (open symbols), tiotropium bromide (A and B), in rabbits exposed to air (blue symbol) or ozone (red symbol). Data are expressed as the mean ± S.D. of percentage increase in baseline R_L_ (A) or percentage decrease in baseline C_dyn_ (B). For the purposes of visualization of the data, the untreated mean curves are duplicated in each panel.

Baseline R_L_ (air vs. ozone: 81 ± 4 vs. 46 ± 2 cmH_2_0.s/l) and C_dyn_ (air vs. ozone: 2.84 ± 0.2 vs. 3.8 ± 0.6 cmH_2_0/ml) was not significantly altered by exposure to this pollutant. Methacholine caused a dose-dependent increase in baseline R_L_ and fall in C_dyn_) in animals exposed to air, which was augmented following 1-hour exposure to ozone (Fig. 5). Airway sensitivity to methacholine, as measured by C_dyn_ PC35 (mean, 95% CI) was significantly increased in rabbits exposed to ozone [air: 3.71 (2.65–3.18 mg/ml) vs. ozone 1.34 (0.91–1.77 mg/ml), *P* < 0.05]. In terms of R_L_ PC50, there was no statistically significant difference in airway sensitivity between air- [2.72 (2.08–3.34 mg/ml)] and ozone-exposed rabbits [1.32 (0.80–1.84 mg/ml].

The muscarinic receptor antagonist tiotropium bromide (*N* = 8), administered 3 hours earlier, suppressed airway obstruction to inhaled methacholine in rabbits exposed to either air or ozone (Fig. 5). From these experiments, a pharmacologically active dose of the muscarinic receptor antagonist was confirmed for the hypertussive model.

#### Effect of Salbutamol or Roflumilast on Ozone-Induced Hypertussive Responses.

**Fig. 6.**
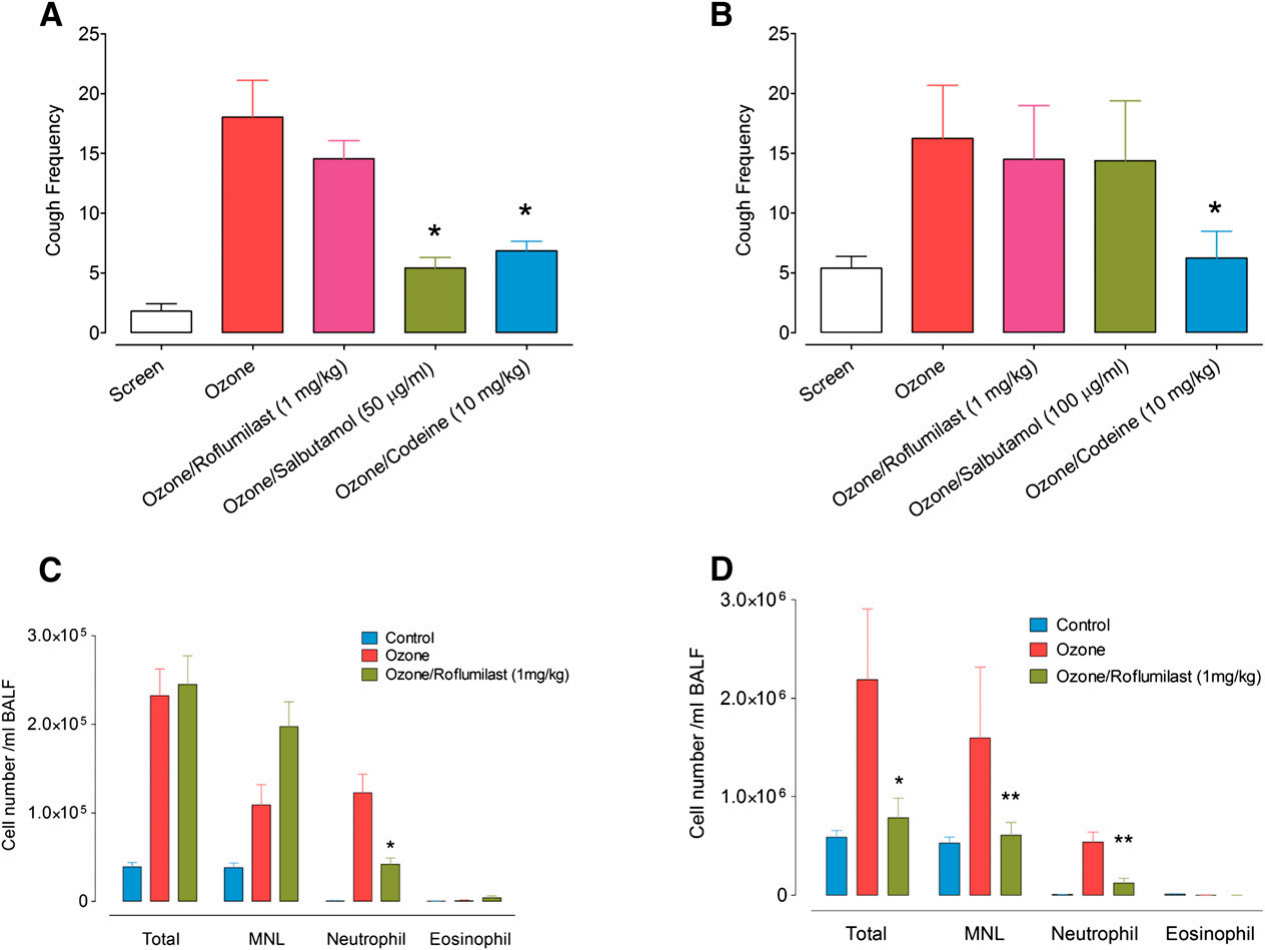
The effect of salbutamol (A, 50 *μ*g/ml; B, 100 *μ*g/ml aerosol), roflumilast (1 mg/kg, i.p.), and codeine (10 mg/kg, i.p.) on the frequency of cough in response to citric acid (30 mM and 0.8 M) in guinea pigs (A) and rabbits (B), respectively, exposed to air or ozone (2 ppm, 30 minutes and 1 hour, respectively). The treatment an animal received at 7-day intervals was randomized to control for sequence effects. Animals were exposed to salbutamol (5 minutes) prior to citric acid challenge and to roflumilast or codeine before ozone exposure. Bars represent the mean, and vertical lines, S.E.M. (*P < 0.05, †*P* > 0.05, compared with ozone/vehicle group; for A and B, *N* = 32 and 16, respectively). The effect of roflumilast on pulmonary leukocyte recruitment in guinea pigs (C) and rabbits (D) following exposure to air or ozone. Animals were exposed to roflumilast (1 mg/kg, i.p.) before ozone exposure. Bars represent the mean, and vertical lines, S.E.M. (*P < 0.05 vs. ozone/vehicle group; guinea pig, *N* = 16; rabbit, *N* = 8). BALF, bronchoalveolar lavage fluid; MNL; mononuclear cells.

Treatment with the *β*_2_-adrenoceptor agonist salbutamol (50 *μ*g/ml, aerosol, 2 minutes at 3 l/min) significantly reduced the hypertussive response to citric acid in guinea pigs previously exposed to ozone [delta change = 12.6 (6.2–19.1), *P* = 0.0004, Fig. 6A]. In contrast, salbutamol (100 *μ*g/ml, aerosol, 2 minutes at 3 l/min) did not significantly alter the ozone-induced hypertussive response to citric acid in rabbits [delta change = 1.9 (−2.6–6.4), *P* > 0.05, Fig. 6B).

Airway obstruction (percent baseline R_L_) induced by 10 mg/ml methacholine in rabbits was reversed by salbutamol (before: 116 ± 12 vs. after: 16 ± 13, *n* = 4). Similarly, airway obstruction induced by 0.64 mg/ml methacholine in guinea pigs was reversed by salbutamol (112 ± 10 vs. 4 ± 13, *n* = 4).

Treatment with the phosphodiesterase 4 inhibitor roflumilast (1 mg/kg, i.p.) did not significantly alter the ozone-induced response to citric acid–induced cough in guinea pigs [delta change = 3.5 (−3.7–10.7), *P* > 0.05, Fig. 6A] or rabbits [delta change = 1.8 (−1.4–4.9), *P* > 0.05, Fig. 6B]. However, the dose of roflumilast used in these studies significantly reduced the recruitment of leukocytes in guinea pigs (Fig. 6C) and rabbits (Fig. 6D) following ozone exposure.

#### The Effect of the TRPA1 Agonist Cinnamaldehyde in Air- or Ozone-Induced Animals.

**Fig. 7.**
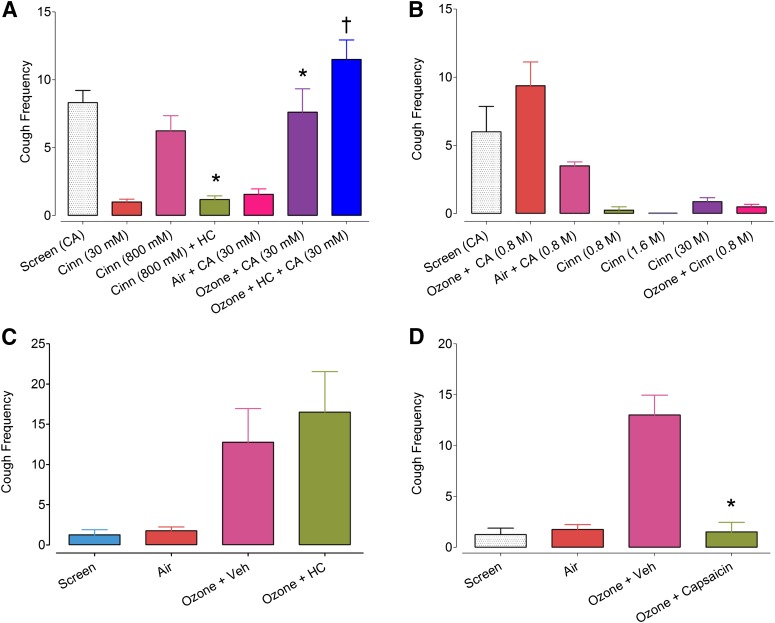
Frequency of cough in response to the TRPA1 agonist cinnamaldehyde (Cinn) in guinea pigs (A), rabbits exposed to air or ozone (2 ppm, 30 minutes and 1 hour, respectively) (B and C), and rabbits chronically treated with capsaicin prior to exposure with ozone and citric acid (CA) (D). Animals were treated with the TRPA1 antagonist HC-030031 (HC; 300 mg/kg) or vehicle (Veh) 30 minutes before exposure to air or ozone. The treatment an animal received at 7-day intervals was randomized to control for sequence effects. Bars represent the mean, and vertical lines, S.E.M. (*P < 0.05 compared with cinnamaldehyde group; *P < 0.05 for comparison between ozone and air with citric acid; †*P* > 0.05 for comparison between ozone+CA and ozone+HC+CA). *N* numbers for (A–D) = 16, 4–8, 4 and 4, respectively).

Cinnamaldehyde (800 mM) elicited a significant cough response in guinea pigs comparable to that achieved with citric acid (100 mM) in air-exposed guinea pigs (Fig. 7A). The TRPA1 antagonist HC-030031 (300 mg/kg, i.p., 1 hour prior to cinnamaldehyde challenge) significantly reduced the frequency of cough in response to cinnamaldehyde. There was a significant increase in cough number [mean (95% CI): 6 (2 – 10) coughs, *P* < 0.05] in response to citric acid in animals exposed to ozone versus air (Fig. 7A). However, this dose of HC-030031 did not significantly alter the cough response to citric acid (30 mM) versus ozone (Fig. 7A).

In contrast to guinea pigs, cinnamaldehyde did not elicit a significant cough response in either air- or ozone-exposed rabbits (Fig. 7B) despite these animals exhibiting a clear hypertussive response to inhaled citric acid. Furthermore, this hypertussive response to citric acid in the rabbit was not inhibited by HC-030031 (*P* > 0.05; Fig. 7C).

The role of TRPV1-positive neurons in hypertussive cough was confirmed in rabbits chronically treated with capsaicin, resulting in a significant reduction in the cough response to citric acid following exposure to ozone (Fig. 7D).

#### The Effect of Known Antitussive Agents on Ozone-Induced Hypertussive Responses.

A range of antitussive agents used to treat cough in humans was also evaluated against ozone-induced hypertussive responses to citric acid in both guinea pigs and rabbits to validate the model. Treatment with the mu-opioid agonist codeine (10 mg/kg, i.p., 5 minutes prior to ozone exposure) significantly suppressed the cough response to citric acid exposed to ozone in guinea pigs [delta change = 11.2 (4.8–17.6), *P* = 0.0012; Fig. 6A] and in rabbits [delta change = 10 (6.25–16.25), *P* < 0.05; Fig. 6B].

**Fig. 8.**
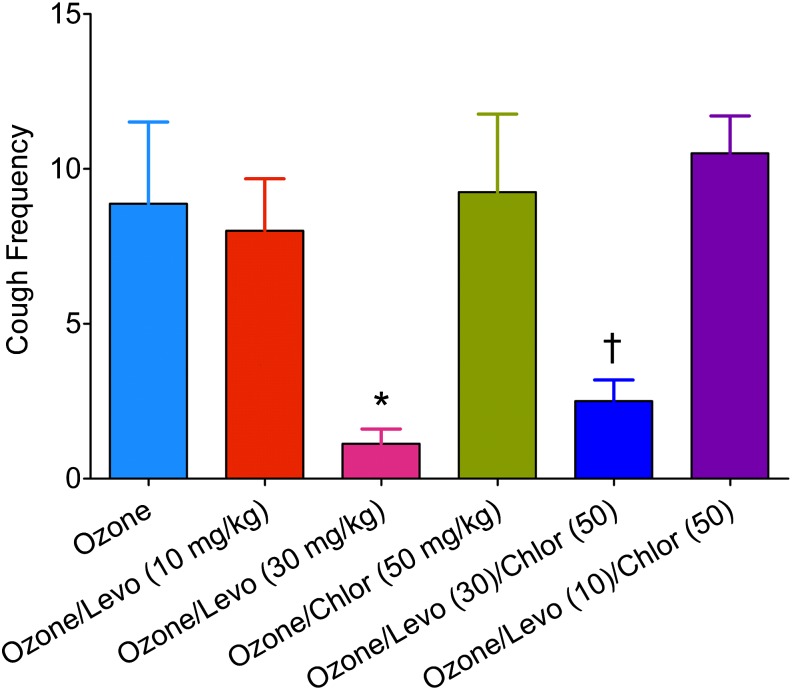
Frequency of cough in response to citric acid in guinea pigs (30 mM) exposed to air or ozone (2 ppm, 30 minutes) following treatment with levodropropizine (Levo; 10 and 30 mg/kg) and chlorpheniramine (Chlor; 50 mg/kg) and in combination (Lev/Chlor; 10/50 and 30/50 mg/kg) before exposure to ozone. The treatment an animal received at 7-day intervals was randomized to control for sequence effects. Bars represent the mean, and vertical lines, S.E.M. [*P < 0.05 compared with ozone/vehicle group and ozone/Levo (10 mg/kg); †*P* > 0.05 compared with ozone/Levo (30 mg/kg, *N* = 8)].

Treatment with a high dose (30 mg/kg, i.p.), but not a low dose, of levodropropizine (10 mg/kg, i.p.) 30 minutes before ozone sensitization significantly reduced the ozone-induced hypertussive response to citric acid [delta change = 6.9 (1.1–8), *P* < 0.05; Fig. 8]. In contrast, treatment with the H1-receptor antagonist chlorpheniramine (50 mg/kg, i.p.) 30 minutes before ozone sensitization did not significantly reduce the ozone-induced hypertussive response (Fig. 8). Furthermore, the combination of levodropropizine and chlorpheniramine (both 30 mg/kg) provided no further effect on the frequency of cough in ozone-exposed rabbits than observed with the high dose of levodropropizine alone (Fig. 8).

## Discussion

We have demonstrated that acute exposure of rabbits and guinea pigs to an inhalation of 2 ppm of ozone can elicit a pronounced hypertussive response to inhaled citric acid in both species. However, significant species differences exist concerning the effect of bronchodilators on this hypertussive cough response. Furthermore, this hypertussive response induced by ozone appears to be independent of the recruitment of inflammatory cells and involves the activation of TRPV1 receptors, but not TRPA1 receptors.

The ozone-induced hypertussive response to citric acid in the rabbit was significantly reduced following chronic treatment of rabbits with capsaicin for 3 days, suggesting the involvement of sensory nerves in this process. The capsaicin protocol we used in these experiments has previously been demonstrated to reduce airway hyper-responsiveness in the rabbit, induced by various inflammatory mediators without influencing the level of sensory neuropeptides (Spina et al., 1991). Unlike the guinea pig, the density of neuropeptide-containing nerves in the rabbit lung is sparse, as in human lungs (Spina et al., 1998). For this reason, we have previously advocated the use of the rabbit to study neural mechanisms in the lung (Keir and Page, 2008). Our results, combined with our previous work, would suggest that this effect of capsaicin is likely due to desensitization of TRPV1 receptors, rather than depletion of sensory neuropeptides per se, and hence functional impairment of the activity of TRPV1-positive neurons, which include C-fibers and a subset of A*δ* fibers (Canning and Spina, 2009). It remains to be established whether the hypertussive response to citric acid is due to altered signaling via TRPV1. The cough induced by citric acid in the rabbit is likely due to the contribution of both acid-sensing ion channels (ASICs) and TRPV1 on afferent nerves. For example, capsaicin can elicit action potentials in bronchial and pulmonary C-fibers in the rabbit (Adcock et al., 2003), and neuropeptide released from peripheral endings of neuropeptide-containing C-fibers can activate rapidly adapting receptors, presumably due to edema in the airways, in this species (Matsumoto et al., 1997). In the guinea pig, citric acid activates ASICs in both nerve types (Kollarik and Undem, 2002).

This suppression of the hypertussive cough response following chronic capsaicin treatment leads to two possible conclusions. First, desensitization/neuronal damage specific to TRPV1-positive nerves following chronic treatment with capsaicin has impaired the ability of citric acid to activate either ASICs or TRPV1 on these nerve fibers, leading to loss in the detection of a cough reflex. Alternatively, following acute exposure to ozone, peripheral sensitization of C-fibers may lead to increased synaptic transmission and processing within the brainstem from action potential generated in both afferent Aδ (via ASIC) and C-fiber (via ASIC and TRPV1) in response to citric acid (Canning and Spina 2009). Ablation of TRPV1-positive nerves therefore leads to a suppression of the hypertussive cough response to citric acid via ASIC on Aδ fibers. It should be recalled that, unlike the guinea pig, the rabbit is relatively refractory to citric acid, and this could reflect a lower C-fiber activity in the rabbit. We did not evaluate the effect of a TRPV1 antagonist on the hypertussive response, since these drugs have been shown to suppress cough in response to citric acid and therefore would also confound interpretation of the exact nature of the involvement of TRPV1 in hypertussive cough (Trevisani et al., 2004).

Furthermore, it is highly unlikely that this effect of capsaicin is due to any anti-inflammatory since in a previous report we found no evidence that capsaicin pretreatment influenced airway inflammation induced by other inflammatory insults in the rabbit (Spina et al., 1991). Furthermore, we also evaluated the effect of the selective phosphodiesterase 4 inhibitor roflumilast n-oxide (Rabe et al., 2005) which, as expected, inhibited ozone-induced airway inflammation, yet failed to inhibit the ozone-induced hypertussive responses to inhaled citric acid. Thus, the ozone-induced hypertussive state in this model is unlikely to be secondary to airway inflammation. It is plausible that ozone causes a local activation/damage of resident pulmonary cells (e.g., airway epithelium, macrophages) to release hyaluronan, an extracellular component of matrix which stimulates Toll receptor 4, (e.g., release of hyaluronan) (Hollingsworth et al., 2004; Williams et al., 2007; Garantziotis et al., 2010; Li et al., 2011) and subsequent downstream signaling, leading to the release of a variety of mediators (e.g., prostaglandins, brain-derived neutrophic factor, TNF*α*, adenosine triphosphate) which are known to sensitize afferent neurons (Canning and Spina, 2009), potentially leading to a hypertussive state.

It has previously been reported that ozone may activate TRPA1 receptors on sensory nerves (Taylor-Clark and Undem, 2010), which could provide a plausible explanation for the ozone-induced hypertussive response. For example, TRPA1 agonists, such as cinnamaldehyde, are known to be protussive in guinea pigs and healthy human subjects (Birrell et al., 2009; Brozmanova et al., 2012). We were able to confirm these findings, albeit at higher doses than has been published in the guinea pig. However, the TRPA1-selective antagonist HC-030031 failed to inhibit the ozone-induced hypertussive response to citric acid in guinea pigs or in rabbits. Interestingly, we were unable to find any functional evidence for constitutive expression of TRPA1 on afferent neurons in the rabbit, unlike the guinea pig, since cinnamaldehyde did not induce cough in this species. As such, we believe our results are not compatible with a major role for TRPA1 in this model, and suggest that ozone is unlikely to cause a hypertussive state via its proposed action at this receptor type.

Recent clinical studies have reported that the long-acting muscarinic receptor antagonist tiotropium bromide may reduce cough in patients with chronic obstructive pulmonary disease (Casaburi et al., 2002; Hasani et al., 2004; Tashkin et al., 2008), and indeed, others have reported experimentally that, in the guinea pig, tiotropium bromide may be antitussive via an additional inhibitory effect on TRPV1 receptors (Birrell et al., 2014). However, although we also documented inhibition of the ozone-induced hypertussive state in guinea pigs following treatment with tiotropium bromide in the present study, we were unable to show any effect of tiotropium bromide on ozone-induced hypertussive responses in the rabbit. This lack of effect was not due to use of an inadequate dose of the muscarinic receptor antagonist, as we used doses clearly capable of inhibiting methacholine-induced bronchoconstriction in this species, confirming previous work in guinea pigs with a related compound, (3*R*)-3-[[[(3-fluorophenyl) [(3,4,5-trifluorophenyl)methyl]amino] carbonyl] oxy]-1-[2-oxo-2-(2-thienyl)ethyl]-1-azoniabicyclo[2.2.2]octane bromide (CHF5407) (Villetti et al., 2010). Interestingly, ozone also induced bronchial hyper-responsiveness to methacholine, and we plan to further investigate this phenomenon in the future.

Therefore, we conclude that the reduction in ozone-induced hypertussive responses in the guinea pig does not reflect a true antitussive effect of this drug class per se, but was secondary to bronchodilation or functional antagonism exhibited by these drugs in this species. This conclusion is further supported by our observations that the *β*_2_-selective agonist salbutamol inhibited the ozone-induced hypertussive response in the guinea pig, but not in the rabbit. In both species, the dose of salbutamol used provided significant bronchoprotection, albeit to a greater extent in the guinea pig. Rabbits express a greater ratio of *β*_1_- to *β*_2_-adrenoceptors (Rugg et al., 1978), whereas salbutamol is 29-fold selective for *β*_2_- than *β*_1_-adrenoceptors (Baker, 2005). Thus, this lack of antitussive effect in the rabbit cannot be simply explained by differences in receptor subtype expression since bronchoprotection was afforded by salbutamol in the rabbit. In contrast, the purported antitussive action of various long-acting *β*_2_-selective agonists in the guinea pig coincided with their bronchoprotective action (Wex and Bouyssou, 2015), although in the case of indacaterol, a protussive action was observed. The authors concluded that the antitussive action of olodaterol and, to a lesser extent, salmeterol and formoterol was attributed to activation of *β*_2_-adrenoceptors on sensory nerves, leading to hyperpolarization of afferent endings. The clinical significance of these findings is difficult to interpret, and although patients with chronic obstructive pulmonary disease demonstrate a lower incidence of symptom scores, which include cough, following treatment with long-acting *β*_2_-adrenoceptor agonists, the interpretation is confounded by the bronchodilator action of this drug class (Pounsford et al., 1985). Clearly, clinical trials with bronchodilators in subjects with idiopathic cough are required to address this issue.

Our study raises some questions as to the predictive value of using the guinea pig to study antitussive drugs, and indeed, neurokinin receptor antagonists, which have been shown to suppress citric acid–induced cough in conscious guinea-pigs (Girard et al., 1995; Yasumitsu et al., 1996; Daoui et al., 1998; El-Hashim et al., 2004), were subsequently demonstrated to not be antitussive in the clinic. Hence, it is difficult to rule out the possibility that, at least in conscious guinea pigs, the antitussive action of the neurokinin receptor antagonists could in part be secondary to a suppression of airway obstruction induced by contraction of airway smooth muscle, edema, and mucus secretion, which together could lead to the activation of A*δ* fibers, which are also implicated in cough (Adcock et al., 2003), rather than a true antitussive effect. Therefore, it remains plausible that the reduction in cough seen clinically following treatment with tiotropium bromide is secondary to bronchodilation, or possibly by an effect on mucus secretion, rather than a true anti-tussive effect.

As part of the validation of ozone-induced hypertussive responses, we also evaluated the effect of codeine, a drug that has been reported to be antitussive in humans (Dicpinigaitis et al., 2014) and to have a significant effect on ozone-induced hypertussive responses in the rabbit (Adcock et al., 2003). Our present results with codeine confirm our earlier work in this model (Adcock et al., 2003) and provide a useful positive control against which to assess other agents. We also saw a significant effect of another clinically effective antitussive, levodropropizine (Dicpinigaitis et al., 2014; Zanasi et al., 2015), but were not able to see any significant antitussive effect of the H1 receptor antagonist chlorpheniramine, despite the fact that this class of drug has been claimed to have modest antitussive effects clinically, albeit not in all studies (Dicpinigaitis et al., 2014).

In the quest for an improved preclinical model of cough, we have described the ability of ozone to induce a hypertussive state in both guinea pigs and rabbits that could provide a convenient model for both the evaluation of the mechanisms underlying the hypertussive state and the evaluation of new drugs. However, our work has highlighted important differences between these two species with respect to the contribution of bronchodilation to the regulation of cough, and suggests that some caution may be required in interpreting experiments with guinea pigs before claiming a true antitussive action of investigational drugs. Furthermore, we have used a crossover approach for such studies to further improve the robustness of the method, and we anticipate that the use of hypertussive responses will aid in the development of new strategies to reduce unwanted cough.

## Abbreviations

ANOVA: analysis of variance
ASIC: acid-sensing ion channel
BAL: bronchoalveolar lavage, CHF5407, (3*R*)-3-[[[(3-fluorophenyl) [(3,4,5-trifluorophenyl)methyl]amino] carbonyl] oxy]-1-[2-oxo-2-(2-thienyl)ethyl]-1-azoniabicyclo[2.2.2]octane bromide
CI: confidence interval
HC-030031: 2-(1,3-dimethyl-2,6-dioxo-1,2,3,6-tetrahydro-7H-purin-7-yl)-*N*-(4-isopropylphenyl)acetamide
PC: provocative concentration
SB-705498: (1-(2-bromophenyl)-3-[(3R)-1-[5-(trifluoromethyl)pyridin-2-yl]pyrrolidin-3-yl]urea)
TRPV1: transient receptor potential cation channel subfamily V member 1
